# The brain, obesity and addiction: an EEG neuroimaging study

**DOI:** 10.1038/srep34122

**Published:** 2016-09-23

**Authors:** Dirk De Ridder, Patrick Manning, Sook Ling Leong, Samantha Ross, Wayne Sutherland, Caroline Horwath, Sven Vanneste

**Affiliations:** 1 Section of Neurosurgery, Department of Surgical Sciences, Dunedin School of Medicine, University of Otago, New Zealand; 2 Section of Endocrinology, Department of Medicine, Dunedin School of Medicine, University of Otago, New Zealand; 3 Department of Human Nutrition, Dunedin School of Medicine, University of Otago, New Zealand; 4 School of Behavioral and Brain Sciences, University of Texas at Dallas, USA

## Abstract

Obesity is among the greatest challenges facing healthcare systems with 20% of the world’s population afflicted. Great controversy exists whether obesity can be regarded as an addictive disorder or not. Recently the Yale Food Addiction Scale questionnaire has been developed as a tool to identify individuals with traits of addiction towards food. Using clinical and source localized EEG data we dichotomize obesity. Brain activity in food-addicted and non-food-addicted obese people is compared to alcohol-addicted and non-addicted lean controls. We show that food addiction shares common neural brain activity with alcohol addiction. This ‘addiction neural brain activity’ consists of the dorsal and pregenual anterior cingulate cortex, parahippocampal area and precuneus. Furthermore, common neural obesity neural brain activity exists as well. The ‘obesity neural brain activity’ consists of dorsal and pregenual anterior cingulate cortex, posterior cingulate extending into the precuneus/cuneus as well as the parahippocampal and inferior parietal area. However food-addicted differ from non-food-addicted obese people by opposite activity in the anterior cingulate gyrus. This food addiction and non-food-addiction obesity dichotomy demonstrates there is at least 2 different kinds of obesity with overlapping network activity, but different in anterior cingulate cortex activity.

Obesity and its associated comorbidities are a major public health challenge facing the modern world. The approximate worldwide prevalence of overweight and obesity is 50% and 20% respectively^1^. This is associated with enormous healthcare related costs, which in the USA has been calculated to be in excess of $215 billion per year^1^. To date, public health strategies have been unsuccessful at preventing the rapid rise in obesity rates^2^, indicating an urgent need to develop effective interventions at both the population and individual level.

Obesity is regarded as a complex disorder in which genetic, physiologic, psychological and environmental factors all interact to produce the obese phenotype. However pathophysiologic subgroups within obese populations have been difficult to identify. It is also likely that effective treatments will only be realized with personalized treatments targeting specific pathophysiologic abnormalities. Although it has long been recognized that homeostatic centers in the brain play a pivotal role in body weight regulation, more recently brain areas similar to those involved in drug addiction have been implicated in food consumption^3^.

Significant controversy exists as to whether the concept of food addiction is plausible, with arguments both in favour and against^3^,^4^. One view considers obesity as a consequence of food addiction^5^, which proposes that certain foods (those high in fat, salt and sugar) are akin to addictive substances insofar as they engage brain systems and produce behavioural adaptations comparable to those engendered by drugs of abuse^4^,^5^. A second view is that food addiction is a behavioural phenotype that is seen in a subgroup of people with obesity and resembles drug addiction^3^,^5^. This view draws on the parallels between the DSM-IV criteria for a substance-dependence syndrome and observed patterns of overeating such as in binge eating^4^. The clinical similarities has led to the idea that obesity and alcohol addiction may share common molecular, cellular and systems-level mechanisms^3^. Arguments in favour of the food addiction-alcohol addiction link have been discussed before^3^,^4^. There exists a (1) clinical overlap between obesity and drug addiction, (2) a shared vulnerability to both obesity and substance addiction, via the *Taq*1A minor (A1) allele of the dopamine receptor D2 (*DRD2*) gene, which has been associated with alcoholism; substance-misuse disorders, including cocaine, smoking and opioid dependence and obesity (3) Analogous neurotransmitter changes have been described consisting of lower levels of striatal dopamine receptors in obese and addicted humans, as well as (4) different brain responses exist to food-related stimuli in obese individuals compared with non-obese controls in functional imaging studies.

All of these arguments have been criticized stating that the vast majority of overweight individuals have not shown a convincing behavioural or neurobiological profile that resembles addiction, and that the enormous inconsistency emerging from a review of the neuroimaging literature suggests that obesity is a highly heterogeneous disorder^4^.

Thus the question arises whether there is indeed a subset of obese people who are food addicted. This understanding could lead to the development of brain-based pathophysiology-specific treatments for subgroups of obese patients. A quantitative and validated psychometric measure of food addiction has recently been developed, the Yale Food Addiction Scale (YFAS)^6^. The content of the Yale Food Addiction Scale (YFAS) is composed of questions based upon substance dependence criteria in the DSM-IV-TR and scales used to assess behavioral addictions, such as gambling, exercise, and sex, including the South Oaks Gambling Screen, the Exercise Dependence Scale, and the Carnes’ Sexual Addiction Screening Tool^6^. For the diagnosis of food addiction, which resembles a diagnosis of substance dependence, criteria were considered met if participants endorsed three or more of the seven criteria of DSM-IV-R as well as at least one of the two clinical significance items (impairment or distress)^6^. These criteria are (1) Substance taken in larger amount and for longer period than intended, (2) Persistent desire or repeated unsuccessful attempt to quit, (3) Much time/activity to obtain, use, recover, (4) Important social, occupational, or recreational activities given up or reduced, (5) Use continues despite knowledge of adverse consequences (e.g., failure to fulfill role obligation, use when physically hazardous, (6) Tolerance (marked increase in amount; marked decrease in effect), (7) Characteristic withdrawal symptoms; substance taken to relieve withdrawal.

The neural correlates for food addiction based on the YFAS criteria have been investigated by means of fMRI in an evoked setting, looking at how the brain of food addicted obese people differs from lean controls in its response to a food stimulus (chocolate milkshake)^7^. Participants with higher vs lower food addiction scores showed greater activation in the dorsolateral prefrontal cortex and the caudate in response to anticipated receipt of food but less activation in the lateral orbitofrontal cortex in response to receipt of food. Furthermore, in a correlation analysis, food addiction scores correlated with greater activation in the anterior cingulate cortex, medial orbitofrontal cortex, and amygdala in response to anticipated receipt of food^7^. This study suggested that similar patterns of neural activation are implicated in addictive-like eating behaviour and substance dependence^7^. Indeed, more reward circuit activation in response to food cues and reduced activation of inhibitory regions in response to food intake was identified^7^.

Craving-related changes in the brain were investigated by cue evoked technique as well with fMRI. Craving related activity was identified in the hippocampus, insula, and caudate, three areas reported to be involved in drug craving as well, supporting the common substrate hypothesis for food and drug cravings^8^.

In a recent study, looking at the neural correlates of food addiction in the resting with source localized EEG, five minutes after a single taste of a chocolate milkshake, patients with three or more food addiction symptoms showed an increase of delta power in the right middle frontal gyrus (Brodmann Area [BA] 8) and in the right precentral gyrus (BA 9), and theta power in the right insula (BA 13) and in the right inferior frontal gyrus (BA 47). Furthermore, compared to controls, patients with three or more food addiction symptoms showed an increase of functional connectivity in fronto-parietal areas in both the theta and alpha band. The increase of functional connectivity was also positively associated with the number of food addiction symptoms^9^. This study suggested that food addiction has similar neurophysiological correlates of other forms of substance-related and addictive disorders suggesting similar psychopathological mechanisms^9^.

The aim of this study was to investigate whether obese people with and without food addiction have a common ‘*obesity neural brain activity’* as well as whether, based on the previous literature, a common ‘addiction neural brain activity’ could be identified between alcohol addicted and food addicted people.

## Methods

### Research subjects

Twenty healthy normal-weight adults and 46 obese participants were included in the study. All participants were recruited from the community by way of newspaper advertisement. In addition, we collected data from 14 individuals who met the criteria for alcohol addiction.

### Procedures

All potential participants attended the research facilities for a screening visit and to provide informed consent. The study protocol was approved by the Southern Health and Disability Ethics Committee at the University of Otago (LRS/11/09/141/AM01) and was carried out in accordance with the approved guidelines. Informed consent was obtained from all participants. Inclusion criteria were male or female participants aged between 20 and 65 years and a BMI 19–25 kg/m^2^ (lean group) or >30 kg/m^2^ (obese group). Participants were excluded if they had other significant co-morbidities including diabetes, malignancy, cardiac disease, uncontrolled hypertension, psychiatric disease (based on question whether they had previously been diagnosed with a psychiatric disease), previous head injury or any other significant medical condition. Obese participants were not receiving any interventions for obesity at the time of the data collection. All participants had anthropometric measurements, physical examination, resting energy expenditure and body composition analysis. Subsequently, those participants who met inclusion criteria attended the clinic after an overnight fast for EEG analysis, blood collection and questionnaire assessments. Inclusion criteria for the alcoholic patients were male and female participants between 20 and 65 years and fulfilling the criteria for alcohol dependence criteria according to the DSM-IVr which was based on a psychiatrist’s evaluation. In addition, they also had to score highly on the obsessive compulsive craving score, have had at least one residential treatment period, previous treatment with at least one anti-craving medication, and at least one outpatient professional healthcare intervention. Patients were excluded if they had psychiatric disorders with psychotic or manic symptoms, previous head injury or any other significant medical condition. This was done by asking patients if they had been previously diagnosed with any psychiatric disease.

Those participants who met inclusion criteria attended after overnight alcohol abstinence for EEG analysis, blood collection and questionnaire assessments.

### Behavioral and lab measures

#### Questionnaires

##### Yale Food Addiction Scale

Each participant completed the Yale Food Addiction Scale which is a is a self-reported standardized questionnaire, based on DSM-IV codes for substance dependence criteria, to identify individuals at high risk for food addiction, regardless of body weight^6^,^10^,^11^. While there is currently no official diagnosis of “food addiction”, the YFAS was created to identify persons who exhibited symptoms of dependency towards certain foods. Foods with addictive potential most notably identified by YFAS include those high in fat and sugar. The YFAS is a psychometrically validated tool consisting of 27 questions that identifies eating patterns that are similar to behaviors seen in classic areas of addiction (2). Using the continuous scoring system scale we calculated an YFAS score out of 7 for each participant (2). A median-split was applied on the YFAS to differentiate the obesity groups. The participants who had a score equal to the median (=3) were excluded from the analysis. Participants with a score lower than the median were assigned to the low YFAS group, i.e. the non-food-addicted obesity group (NFAO) while those with a score higher than the median were assigned to the high YFAS group, i.e. the food-addicted obesity group (FAO).

*Numeric rating scales* (NRS) from 0 to 10 measuring hunger (How hungry do you feel?); satisfaction (How satisfied do you feel?); fullness (How full do you feel?); appreciation (How much do you think you can eat right now?); and food desire/craving (Would you like to eat something right now?).

##### BIS/BAS

The behavioral inhibition system/behavioral approach system (BIS/BAS) scales were developed to assess individual differences in the sensitivity of two general motivational systems which underlie behavior^12^. A BIS is said to regulate aversive motives, in which the goal is to move away from something unpleasant. A BAS is believed to regulate appetitive motives, in which the goal is to move toward something desired.

##### DEBQ

Participants completed a copy of the Dutch Eating Behavior Questionnaire (DEBQ) by indicating the extent to which they eat for emotional reasons, external reasons and restraint^13^.

##### BES

The Binge eating scale (BES) is a questionnaire assessing the presence of certain binge eating behaviors which may be indicative of an eating disorder^14^.

##### Food Awareness

Food awareness is quantified by a subscale of the mindful eating questionnaire^15^ and measures affective sensitivity of the internal states and organoleptic awareness (i.e. conscious appreciation of the effects of food on each of the senses).

#### Laboratory and visit measurements

Venous blood samples were sent to the laboratory of Dunedin Public Hospital for measurement of glucose, lipids and liver function by standard methods. Body composition was measured using bioelectrical impedance analysis (BIA) (Tanita MC-780 Multi Frequency Segmental Body Composition Analyser). Resting energy expenditure was measured by indirect calorimetry (Fitmate, COSMED).

#### Group comparisons

A median-split was applied on the YFAS to differentiate the obesity groups. Eight participants had a score equal to the median (=3) and were excluded from the analysis. Participants with a score lower than the median were assigned to the low YFAS group, i.e. the non-food-addicted obesity group (NFAO) while those with a score higher than the median were assigned to the high YFAS group, i.e. the food-addicted obesity group (FAO). Technically speaking only 3 participants really met the criteria for food addiction, i.e. three or more of the seven criteria of DSM-IV-R as well as at least one of the two clinical significance items (impairment or distress) (Gearhardt, Corbin *et al.*^6^).

A comparison was made between the lean, low YFAS and high YFAS groups for the different questionnaires using a MANOVA. As dependent variables all the questionnaires were included in one single model as listed in Table 1. The independent variable was the group (lean, low YFAS and high YFAS). A correction for multiple comparisons was applied using a Bonferroni correction (p < 0.05) to make a comparison between the three different groups. We included the variable age as a covariate to control our findings for age.

We performed a study analysing biochemical and clinical data, as well as food and obesity related questionnaires (see Tables 1 and 2) complemented by resting state brain EEG activity in a group of obese (BMI > 30 kg/m^2^) people (n = 38) with low (n = 18) and high (n = 20) YFAS scores and compared them to a group of lean non-addicted controls (n = 20), using source localized EEG recordings.

In addition, to verify whether a high YFAS score indeed reflects an addictive phenotype we compared the high and low YFAS groups to a group of intractable alcohol addicted people (n = 13), looking for a common neural addiction network, as well as the neural substrates of food and alcohol craving.

### Correlation between food addiction and binge eating

In view of the known correlation between food addiction and Binge Eating (BES > 17)^11^,^16^ a correlation analysis was performed between YFAS and BES. Furthermore, the BES group was divided in a high BES (>17) and low BES group and this was related to the YFAS group (high versus low YFAS).

### Electrical neuroimaging

#### EEG Data collection

EEG data were obtained as a standard procedure. Recordings were obtained in a fully lighted room with each participant sitting upright on a small but comfortable chair. The actual recording lasted approximately five minutes. The EEG was sampled using Mitsar-201 amplifiers (NovaTech http://www.novatecheeg.com/) with 19 electrodes placed according to the standard 10–20 International placement (Fp1, Fp2, F7, F3, Fz, F4, F8, T7, C3, Cz, C4, T8, P7, P3, Pz, P4, P8, O1, O2). Participants abstained from alcohol consumption 24 hours prior to EEG recording and from caffeinated beverages on the day of recording in order to avoid alcohol-induced changes in EEG^17^ or a caffeine-induced alpha power decrease^18^,^19^. The vigilance of participants was monitored by EEG parameters such as the slowing of alpha rhythm or the appearance of spindles as drowsiness is reflected in enhanced theta power^20^. Impedances were checked to remain below 5 kΩ. Data were collected eyes-closed (sampling rate = 500 Hz, band passed 0.15–200 Hz). Off-line data were resampled to 128 Hz, band-pass filtered in the range 2–44 Hz and subsequently transposed into Eureka! software^21^, plotted and carefully inspected for manual artifact-rejection. All episodic artifacts including eye blinks, eye movements, teeth clenching, body movement, or ECG artifact were removed from the stream of the EEG. In addition, an independent component analysis (ICA) was conducted to further verify if all artifacts had been excluded. To investigate the effect of possible ICA component rejection, we compared the power spectra with two approaches: (1) after visual artifact rejection only, and (2) after additional ICA component rejection. The mean power in delta (2–3.5 Hz), theta (4–7.5 Hz), alpha1 (8–10 Hz), alpha2 (10–12 Hz), beta1 (13–18 Hz), beta2 (18.5–21 Hz), beta3 (21.5–30 Hz) and gamma (30.5–44 Hz) bands^22^,^23^,^24^ did not show a statistically significant difference between the two approaches. We were therefore confident in reporting the results of two-step artifact correction data, namely visual artifact rejection and additional independent component rejection. Average Fourier cross-spectral matrices were computed for all eight bands.

#### Source localization

Standardized low-resolution brain electromagnetic tomography (sLORETA^25^,^26^) was used to estimate the intracerebral electrical sources that generated the seven group BSS components. As a standard procedure a common average reference transformation^25^ is performed before applying the sLORETA algorithm. sLORETA computes electric neuronal activity as current density (A/m2) without assuming a predefined number of active sources. The solution space used in this study and associated leadfield matrix are those implemented in the LORETA-Key software (freely available at http://www.uzh.ch/keyinst/loreta.htm). This software implements revisited realistic electrode coordinates (Jurcak *et al.* 2007) and the lead field produced by Fuchs *et al.*^27^ applying the boundary element method on the MNI-152 (Montreal neurological institute, Canada) template of Mazziotta *et al.*^28^,^29^. The sLORETA-key anatomical template divides and labels the neocortical (including hippocampus and anterior cingulate cortex) MNI-152 volume in 6,239 voxels of dimension 5 mm^3^, based on probabilities returned by the Demon Atlas^30^,^31^. The co-registration makes use of the correct translation from the MNI-152 space into the Talairach and Tournoux^32^ space^33^.

#### Correlation analysis

The methodology used for the sLORETA correlations is non-parametric. It is based on estimating, via randomization, the empirical probability distribution for the max-statistic, under the null hypothesis comparisons^34^. This methodology corrects for multiple testing (i.e., for the collection of tests performed for all voxels, and for all frequency bands). Due to the non-parametric nature of the method, its validity does not rely on any assumption of Gaussianity^34^. sLORETA statistical contrast maps were calculated through multiple voxel-by-voxel comparisons. The significance threshold was based on a permutation test with 5000 permutations. Correlations are calculated for the alcohol, low YFAS and high YFAS groups with craving, hunger, fullness and awareness scales.

#### Conjunction analysis

In addition to the group comparison between low YFAS and high YFAS, high YFAS and alcohol addicted participants we also conducted a conjunction analysis^35^,^36^,^37^,^38^. A conjunction analysis identifies a ‘common processing component’ for two or more tasks/situations by finding areas activated in independent subtractions^35^,^36^,^37^,^38^. Friston *et al.*^36^ also indicated that although general conjunction analysis is used in a within group condition, it can also be applied between groups and was applied in some recent papers^39^,^40^. We opted to subtract images of the lean group from the low YFAS and high YFAS, high YFAS and alcohol addicted groups so that only pathological activity (activity that deviated from the healthy subjects) remains for low YFAS and high YFAS, high YFAS and alcohol addicted group separately. Based on the images of both low YFAS and high YFAS, high YFAS and alcohol addicted, we conducted a conjunction analysis to see what pathological activity they have in common.

## Results

### Behavioral measures

#### YFAS

Comparison between the lean, low and high YFAS shows a significant difference (*F* = 104.18, *p* < 0.001) indicating that the lean group and the low YFAS do not differ from each other, but that both groups differ from the high YFAS group (Table 3). When we look at the different subscales of the YFAS, food overuse, time spent on food, social withdrawal, withdrawal symptoms and food related are the subscales the differentiate high YFAS from low YFAS subjects. However, the high YFAS group does not differ from the low YFAS and lean group for the subscales persistent use despite adversity and tolerance. On none of the subscales do low YFAS subjects differ from the lean subjects. Table 3 provides a detailed overview.

### Correlation between food addiction and binge eating

The YFAS score for the total group correlated with the BES score (r = 0.50, p < 0.01) (Table 4). For the low YFAS group no significant correlation was found (r = 0.18, p < 0.05) (Table 4), for the high YFAS group a significant correlation was found (r = 0.56, p < 0.05) (Table 4).

#### Demographics, anthropometric and laboratory measures

A comparison between the low and high YFAS groups shows a common phenotype. Both groups cannot be separated based on biochemical analysis (*F* = 0.89, *p* = 0.572), vital signs (*F* = 0.75, *p* = 0.532), weight and other anthropometric measures (*F* = 1.17, *p* = 0.342) including body fat composition (*F* = 0.66, *p* = 0.684), resting energy expenditure (*F* = 0.77, *p* = 0.387). Both obese groups were significantly different from the lean group. The alcohol addicted patients have a normal body weight, height and BMI. Their craving score was 8.32/10 and their alcohol use disorder identification test (audit) score 36.21 (normal <20). See Table 2 for overview.

#### Questionnaires

Both the low and high YFAS group report that they have less hunger that the lean group. The high YFAS group reports that they feel fuller than the low YFAS and lean group. No significant differences were demonstrated for satisfaction, appreciation and food desire. On the BIS/BAS questionnaire, the high YFAS group reports a higher score than the low YFAS and lean group on the BIS, but not on the BAS. On the three different subscales of the DEBQ a significant effect was obtained. For the subscale ‘restrained’ both the low YFAS and high YFAS group reported a higher score in comparison to the lean group, but do not differ from each other. The subscale ‘external’ indicates that high YFAS subjects have a higher score than both the Low YFAS and lean subjects, but that the low YFAS group has a lower score than both the lean and high YFAS group. The ‘emotional’ subscale shows a difference between the high YFAS group and both the low YFAS and lean subjects. In addition, the high YFAS group has a higher score on Binge eating and Food awareness in comparison to the low YFAS and lean group. For Food awareness a significant difference was also obtained between the low YFAS group and lean group. Table 3 shows a summary of the results. In addition Table 4 shows the correlation between the different questionnaires for the whole obese group, the low and high YFAS, separately.

### Electrical neuroimaging

#### Correlation analyses

##### Whole group

**Figure 1:**
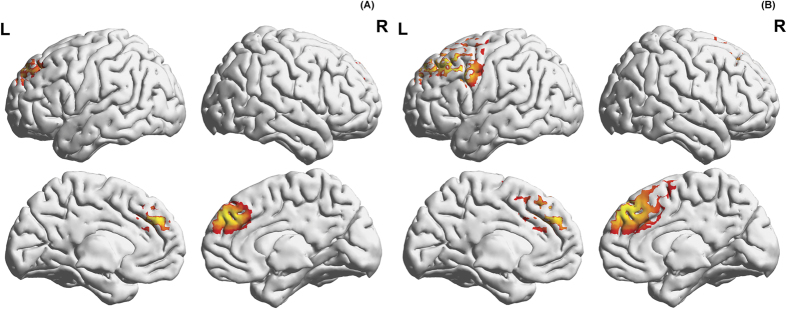
A whole brain correlation analysis and the YFAS revealed a significant positive correlation with the (A) rostral anterior cingulate cortex (rACC) for the theta (r = 0.23, *p* = 0.041) and the (B) beta3 (r = 0.22, *p* = 0.041) frequency bands.

A whole brain correlation analysis and the YFAS revealed a significant positive correlation with the rostral anterior cingulate cortex (rACC) for the theta (r = 0.23, *p* = 0.041) and the beta3 (r = 0.22, *p* = 0.041) frequency bands (Fig. 1).

##### Low YFAS group

**Figure 2:**
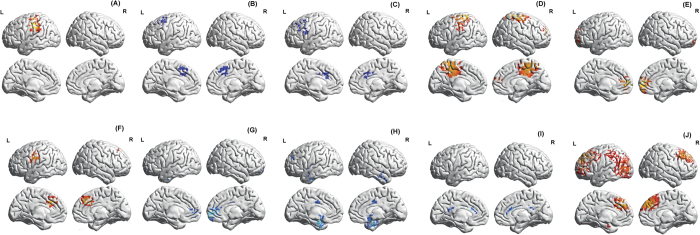
(**A**) Correlation analysis in non-food addicted obese people. Hunger scores correlate positively with theta resting state EEG activity in posterior insula as well as left somatosensory cortex (r = 0.69, p = 0.0007). (**B**) Correlation analysis in non-food addicted obese people. Hunger scores correlate negatively with beta1 resting state EEG activity in dACC (r = −0.49, p = 0.019). (**C**) Correlation analysis in non-food addicted obese people. Hunger scores correlate negatively with beta2 resting state EEG activity in rACC and left insula (r = −0.48, p = 0.022). (**D**) Correlation analysis in non-food addicted obese people. Perception of fullness correlates positively (red) with beta 3 activity in the PCC, extending to the precuneus and somatosensory cortex (r = 0.52, p = 0.013). (**E**) Correlation analysis in non-food addicted obese people. Perception of fullness correlates positively (red) with gamma activity in the pgACC (r = 0.61, p = 0.004). (**F**) Correlation analysis in non-food addicted obese people. Food awareness correlates positively with theta activity in the rACC and somatosensory cortex (r = 0.44, p = 0.034). (**G**) Correlation analysis in non-food addicted obese people. Food awareness correlates negatively (blue) with beta1 activity in the pgACC (r = −0.90, p < 0.00001). (**H**) Correlation analysis in non-food addicted obese people. Food awareness correlates negatively (blue) with beta2 activity in the dACC and the sgACC extending into the amygdala (r = −0.73, p = 0.0003). (**I**) Correlation analysis in non-food addicted obese people. Food awareness correlates negatively (blue) with gamma activity in the dACC and PCC (r = −0.61, p = 0.004). (**J**) Correlation analysis between food craving (=hunger) scores in high YFAS group and gamma band current density (r = 0.56, p = 0.005).

A correlation analysis for the whole brain and the *hunger score* revealed a significant effect for both theta and beta1 and beta2 frequency band. Hunger scores correlate positively with theta resting state EEG activity in posterior insula as well as left somatosensory cortex (r = 0.69, *p* = 0.0007) (Fig. 2A) and correlates negatively with beta1 resting state EEG activity in dorsal anterior cingulate cortex (dACC) (r = −0.49, *p* = 0.019) (Fig. 2B). A negative correlation for beta2 resting state EEG activity in rostral anterior cingulate cortex (rACC) and left insula (r = −0.48, *p* = 0.022) is also found (Fig. 2C). No significant effects were for the delta, alpha1, alpha2, beta3 and gamma frequency band. A positive correlation was obtained between the *perception of fullness* and beta 3 activity in the posterior cingulate cortex (PCC), extending to the precuneus and somatosensory cortex (r = 0.52, *p* = 0.013) (see Fig. 2D) and with gamma activity in the pregenual anterior cingulate cortex (pgACC) (r = 0.61, *p* = 0.004) (Fig. 2E). A positive correlation was obtained between *food awareness* and theta activity in the rACC and somatosensory cortex (r = 0.44, *p* = 0.034) (Fig. 2F). A negative correlation was obtained with beta1 activity in the pgACC (r = −0.90, p < 0.00001) (Fig. 2G). In addition, a negative correlation was demonstrated with beta2 activity in the dACC and the subgenual anterior cingulate cortex (sgACC) extending into the amygdala (r = −0.73, *p* = 0.0003) (Fig. 2H). Furthermore, a negative correlation was found (blue) with gamma activity in the dACC and PCC (r = −0.61, *p* = 0.004) (Fig. 2I). No other significant effects were obtained. No effect was identified between brain activity and the hunger scale for the low YFAS group.

##### High YFAS group

A significant correlation was identified between *hunger scores* and gamma band current density in the rACC extending into the dorsal medial prefrontal cortex (dmPFC) (r = 0.56, *p* = 0.005) (Fig. 2J). No significant effect was identified for the delta, theta, alpha1, alpha2, beta1, beta2 and beta3 frequency bands. No significant correlations existed between brain activity and the hunger, fullness and awareness scales.

##### Alcohol addiction group

**Figure 3:**
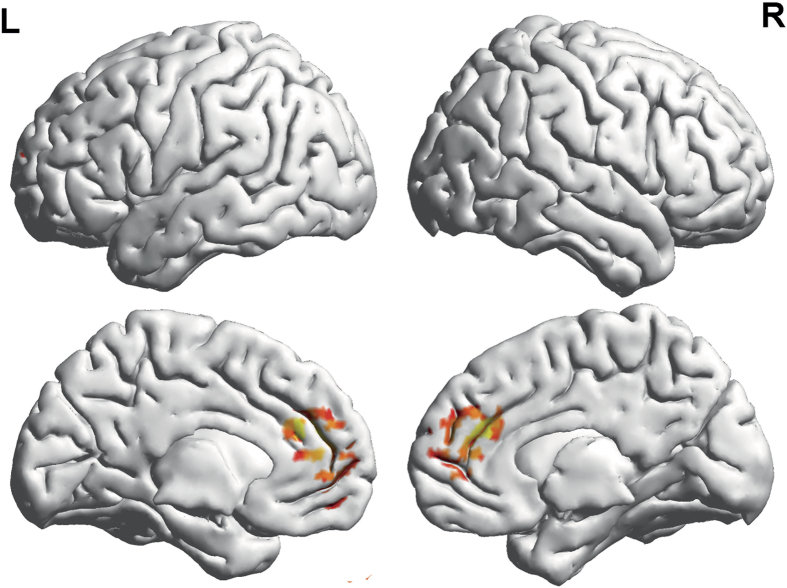
Correlation analysis between alcohol craving scores and gamma band current density (r = 0.72, p = 0.002).

A significant correlation was identified between alcohol craving scores and gamma band current density for the rACC extending into the dmPFC (r = 0.72, *p* = 0.002) (Fig. 3).

#### Conjunction analysis

**Figure 4:**
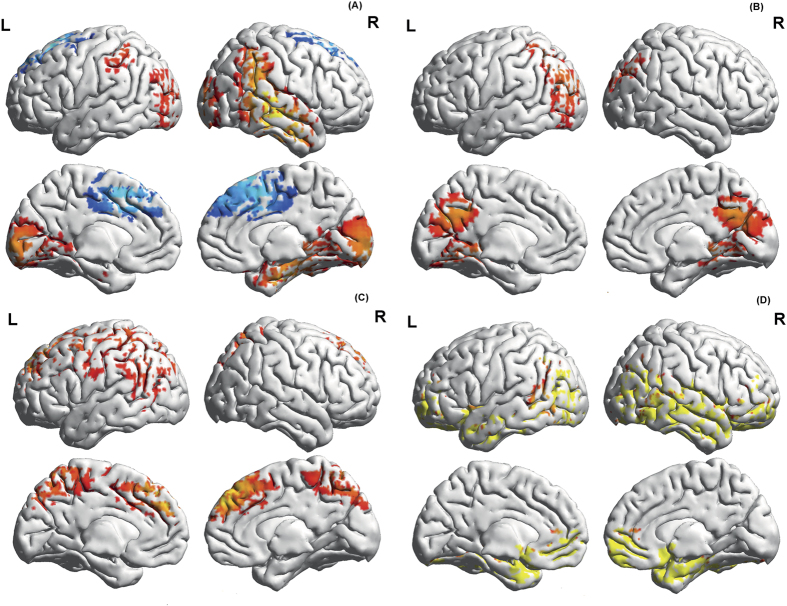
(**A**) Conjunction analysis of beta2 band resting state activity between food addicted obese people (high YFAS) and non-food addicted obese people (low YFAS). Red represents significant deviation from lean healthy non-addicted controls common for both obese groups, blue represents significant but opposite deviation from activity in healthy non-addicted controls in the 2 groups, i.e. blue signifies opposite (Z = −2.03, p = 0.021), red signifies same sign (Z = 2.92, p = 0.0016). (**B**) Conjunction analysis of gamma band resting state activity between food addicted obese people (high YFAS) and non-food addicted obese people (low YFAS) (Z = 1.99, p = 0.023). (**C**) Conjunction analysis between high YFAS obese group and alcohol addiction group for alpha1 activity. Common alpha1 activity for food and alcohol addiction is noted in ACC/dmPFC and precuneus (Z = 2.24, p = 013). (**D**) Conjunction analysis between high YFAS obese group and alcohol addiction group for alpha2 activity. Common alpha2 activity for food and alcohol addiction is noted in sgACC and OFC as well as temporal lobe (fusiform/parahippocampal area) (Z = 2.78, p = 0.003).

A conjunction analysis of resting state activity between high and low YFAS groups shows beta2 activity in the sgACC, pgACC, parahippocampal area, right inferior parietal and midtemporal areas (Z = 1.99, *p* = 0.023) (Fig. 4A) and gamma activity in the PCC extending into precuneus and cuneus (Z = 1.99, *p* = 0.023) (Fig. 4B). Anti-correlated activity in the beta2 frequency was identified in the rACC/dmPFC areas between high YFAS and low YFAS groups (Z = −2.03, *p* = 0.021) (Fig. 4A).

A conjunction analysis between the high YFAS obese group and alcohol addiction group showed a significant effect for the alpha1 frequency band in ACC/dmPFC and precuneus (Z = 2.24, *p* = 0.013) (Fig. 4C) and for alpha2 activity in sgACC and orbitofrontal cortex (OFC) as well as temporal lobe (fusiform/parahippocampal area) (Z = 2.78, *p* = 0.003) (Fig. 4D). No significant effect was seen between the low YFAS groups and the alcohol addiction group.

## Discussion

These results suggest that a high YFAS score does represent an addictive state. The conjunction analysis demonstrated that the high YFAS group and the alcohol addiction group share common pathological brain activity, not present in the low YFAS group. The visualized neural substrate is considered pathological as it is controlled for in both high YFAS and alcohol addicted groups by subtraction of brain activity from a lean non-addicted healthy control group. This pathological ‘addiction brain activity’ involves the anterior cingulate cortex/dorsal medial prefrontal cortex, pregenual anterior cingulate cortex extending into the medial orbitofrontal cortex (mOFC), parahippocampal area and precuneus, brain areas which can be modulated by pharmacological or cognitive based addiction treatments^41^. A previous fMRI study showed that YFAS scores correlated with cue evoked activity in the rACC and mOFC^7^ suggesting that these brain areas are responsive to food cues. Our results indicate that they are also more active in the resting state in contrast to a previous LORETA EEG resting state study^9^. Thus alcohol and food addiction could, apart from cellular, genetic and behavioural aspects^3^, also share a common neurophysiological substrate at a macroscopic brain activity level.

Both YFAS groups, however, have a common phenotype, obesity, and cannot be separated based on biochemical analysis, vital signs, weight and other anthropometric measures including body fat composition, resting energy expenditure, nor on food related rating scores except for fullness perception (Table 2). This clinical similarity is reflected in common neurobiological ‘obesity brain activity’ shared by the low and high YFAS groups. A conjunction analysis (controlled for lean) showed common pathological beta activity in the subgenual and pgACC, with gamma activity in the PCC extending into precuneus and cuneus, and combined with beta activity in parahippocampal area and right inferior parietal and midtemporal area. These areas essentially constitute the default mode network, which is involved in processing self-referential and bodily sensations information^42^. However, it is interesting that different parts of the default mode network are processing information at different frequencies. It has been suggested that the default mode network consists of 3 subnetworks^43^. One part consists of the pgACC/vmPFC and is a critical element in a network of areas that receive sensory information from the external world and the body, and acts as a sensory-visceromotor link concerned with social behavior, mood control, and motivational drive^43^. This part in obese people oscillates at beta activity, which is involved in sensory predictions^44^ and status quo processing^45^. When integrating this into a recently developed concept of behavioural changes^46^ in which the pgACC calculates the reliability of the current behaviour, this hypothetically could suggest that in obese people the pgACC calculates that the obese state is the accepted reference. The PCC/Precuneus oscillates at gamma activity. Gamma activity has been related to prediction errors, or in other words, change, and the PCC/precuneus is the main hub from the self-referential^47^,^48^ default mode network. It can be hypothesized that the PCC/Precuneus resets the references, i.e. controls allostasis^49^, through predictive reference resetting^49^. Allostasis has been implicated in addiction^50^, as well as obesity (food addiction)^49^. In the parahippocampal area and right inferior parietal and midtemporal area beta and gamma oscillations are present. The parahippocampal are is involved in contextual processing^51^,^52^, whereas the right inferior parietal area is involved in multimodal sensory integration center^53^. Beta/gamma coupling has been linked to omitted stimuli^44^. One could speculate that the beta and gamma activity in these areas is related to not processing (omitted food derived stimuli) in the multimodal sensory area and not putting it into a context. Thus in obese people food stimuli could hypothetically be processed in a decontextualized framework,. i.e. irrespective of the context food might be appetitive. On the other hand, significant differences were also identified between the low and high YFAS groups. The conjunction analysis between low YFAS and high YFAS groups demonstrated pathological anti-correlated resting state beta activity in the rACC/dmPFC. This difference is even more striking in the correlation analyses with hunger. Increasing hunger is correlated to increasing gamma activity in the rACC/dmPFC in the high YFAS group, similar to the rACC area correlated to increasing craving in alcohol addiction (Fig. 1 middle, S1C-D). The same area is activated by food cues, putatively generating craving, in people with higher YFAS scores in a fMRI study^7^. By contrast, in the low YFAS group hunger demonstrated a negative correlation with activity in the same rACC area. Previous studies have shown that the rACC is implicated in alcohol craving^54^, and both legal and illicit drug craving^55^. Our finding suggests that it is involved in food craving as well. Differences, although non-significant, in activity in the ACC between obese individuals with higher (>3) versus lower (≤2) food addiction symptoms has previously been reported^9^. The findings from this study may explain why previous neuroimaging studies in obesity have yielded conflicting results.

The ACC has been coined the most interesting part of the brain^56^ because of its many proposed functions., These include salience attribution^57^, Bayesian prediction error processing^58^, representation of the requirements needed for maintaining homeostatic balance^59^, and driving appropriate behavioural responses^60^. This study suggests that in the high YFAS group, there is increased salience attached to food, stimulating an urge to eat^60^.

Hunger in the NFAO group correlates positively with increasing theta activity in the left posterior insula, an area that processes both somatosensory and visceral sensory input, and the left caudal part of the somatosensory cortex, which processes taste as well as intra-abdominal sensory information^61^,^62^. In contrast, hunger correlates negatively with beta activity in left anterior insula, which is involved in processing of affective information from the posterior insula via the autonomic nervous system^62^. This suggests that sensory and affective processing of visceral information in the insula is dissociated in this group. It is tempting to speculate that resistance to homeostatic signals could be responsible for this effect. Further studies are required to investigate this possibility.

How could opposite pathological resting state activity in the dACC result in the same obese phenotype? Even though no explanation yet exists, it is tempting to speculate that a Bayesian brain mechanism might be involved, as this area has been linked to Bayesian learning and prediction error processing^58^,^63^. In the high YFAS group a prediction error calculation problem might be driving an urge for food intake leading to obesity^64^, analogous to what has been suggested for alcohol and other addictions^65^. However, in the low YFAS group, we hypothesize inadequate visceral signals result in an erroneous prediction calculation.

It is known that food addiction and binge eating correlate highly (r = 0.78) (Imperatori, Innamorati *et al.* 2014) and that the association between food addiction and psychopathology is mediated by binge eating in a clinical population (Imperatori, Innamorati *et al.* 2014). And indeed we see a correlation between YFAS and BES scores. However due to the low number of real food addicted people (n = 3) and real binge eaters (n = 2), this study cannot confirm this finding when further analyzed. Indeed, when brain activity was correlated with the hunger, satisfaction, fullness, appreciation and food desire score, in both low and high YFAS groups these scores did not correlate with the BES score. This is a weakness of this study. However, it is of interest that in a group without diagnosed psychopathology a neurophysiological difference can be found between low and high YFAS, which is not identified in the intermediate group. This suggests that even though this group with high YFAS might not represent a representative sample of psychopathologically food addicted people, that in a group without diagnosed psychiatric disease there are still differences between low and high YFAS, and that there exists a group without psychopathology that still has common electrophysiological features with typical addiction, in this case alcohol addiction.

A weakness of the study is that the EEG findings might be merely correlational. Yet for the overlapping ‘addiction neural activity’, between alcohol and food addiction there is some preliminary evidence that the role of the dACC in craving might be causal. Indeed, in a case report using a double cone TMS targeting the dACC it was shown that rTMS could induce a temporary (2–3 weeks) reduction in alcohol craving^66^. Furthermore, in a subsequent case report an electrode was implanted on the dACC of an alcohol addicted patient for a more permanent solution for his alcohol addiction, with a more permanent positive result^67^. This suggests that the dACC might indeed be involved in encoding craving in general, as suggested by a previous meta-analysis looking at the neural correlate of craving across different substances of abuse^55^.

Another weakness of the study is that only an indirect measure for specific food craving was used, i.e. food desire (Would you like to eat something right now?). Even though food craving is an intense desire to obtain and consume food, usually food craving is an intense desire to consume a specific food (e.g. very commonly chocolate), and is different from normal hunger.

A third limitation of this study is the low resolution of the source localization inherently resulting from a limited number of sensors (19 electrodes) and a lack of subject-specific anatomical forward models. This is sufficient for source reconstruction but results in greater uncertainty in source localization and decreased anatomical precision, and thus the spatial precision of the present study is considerably lower than that of functional MRI. Nevertheless, the tomography sLORETA has received considerable validation from studies combining LORETA with other more established localization methods, such as functional Magnetic Resonance Imaging (fMRI)^68^,^69^, structural MRI^70^, Positron Emission Tomography (PET)^71^,^72^,^73^ and was used in previous studies to detect for example activity in the auditory cortex^74^,^75^,^76^. Further sLORETA validation has been based on accepting as ground truth the localization findings obtained from invasive, implanted depth electrodes, in which case there are several studies in epilepsy^77^,^78^ and cognitive ERPs^79^. It is worth emphasizing that deep structures such as the anterior cingulate cortex^80^, and mesial temporal lobes^81^ can be correctly localized with these methods. However, further research could improve spatial precision and accuracy by using high-density EEG (e.g., 128 or 256 electrodes) and subject-specific head models, and MEG recordings.

In conclusion, we demonstrate that in obese individuals, despite identical phenotypic characteristics, at least two neurobiological mechanisms exist that are pathophysiologic. The most salient difference between these two obese groups relates to opposite activity of the dACC. There is also striking similarity between the food and alcohol addicted groups suggesting that a high YFAS score does indicate an addictive disorder related to food and with similar neurobiological processes to alcohol addiction. Our results also suggest that treatments for obesity, such as medication or neuromodulation, should be individualised based on the underlying neurobiological pathophysiology.

## Additional Information

**How to cite this article**: De Ridder, D. *et al.* The brain, obesity and addiction: an EEG neuroimaging study. *Sci. Rep.* **6**, 34122; doi: 10.1038/srep34122 (2016).

## Figures and Tables

**Table 1:** Demographics, anthropometric and laboratory measures for the lean and obese groups.

	Lean	Obese	
	(n = 20)	Low YFAS (n = 18)	High YFAS (n = 20)
Age	37.95 (11.51)	50.56 (9.12)	47.40 (14.37)
Gender	♂ 8 ♀ 12	♂ 2 ♀ 16	♂ 2 ♀ 18
Body weight (kg)	67.60 (9.30)	103.81 (18.91)	106.69 (21.38)
Height (cm)	172.01 (10.38)	166.08 (6.90)	163.91 (6.26)
BMI	22.76 (2.11)	37.67 (6.64)	39.56 (6.50)
Systolic BP	116.85 (10.78)	129.41 (10.34)	134.35 (17.44)
Diastolic BP	75.00 (6.29)	82.88 (7.87)	83.00 (6.37)
Heart rate	65.05 (12.13)	71.47 (9.61)	73.85 (9.26)
Waist	78.75 (5.81)	112.56 (15.17)	118.55 (15.96)
Resting energy expenditure	1625.40 (289.67)	1686.53 (332.69)	1768.40 (234.10)
% body fat	22.93 (8.33)	44.79 (8.45)	46.57 (5.69)
Fat mass	15.11 (5.21)	47.29 (15.94)	50.04 (13.37)
Fat free mass	52.18 (11.16)	56.24 (8.16)	56.48 (11.31)
% trunk fat	21.56 (7.37)	42.84 (7.35)	44.43 (4.81)
Trunk fat mass	7.87 (2.76)	23.60 (6.94)	24.79 (5.75)
Trunk fat free mass	29.07 (5.72)	30.68 (4.08)	30.62 (5.27)
Cholesterol (mmol/L)	4.66 (1.01)	5.56 (1.01)	5.59 (0.92)
Triglycerides (mmol/L)	0.94 (0.40)	1.29 (0.53)	1.37 (0.64)
HDL (mmol/L)	1.57 (0.34)	1.39 (0.28)	1.40 (0.27)
LDL (mmol/L	2.66 (0.83)	3.58 (0.92)	3.58 (0.75)
GGT (U/L)	15.35 (9.78)	34.11 (28.55)	25.95 (13.92)
ALT (U/L)	17.85 (7.67)	27.00 (17.41)	24.30 (11.06)
AST (U/L)	21.45 (5.30)	26.06 (24.97)	22.25 (7.04)
Glucose (mmol/L)	—	5.06 (0.42)	5.17 (0.50)

**Table 2:** Questionnaire analyses: Mean scores and Standard deviations.

	Lean	Obesity		
	(n = 20)	Low YFAS (n = 18)	High YFAS (n = 20)	
Hunger	5.35 (2.41)^a^	3.50 (3.05)^b^	3.21 (1.90)^b^	**F** = **7.97, p** = **0.001**
Satisfied	2.35 (1.66)	2.94 (1.86)	3.00 (1.45)	F = 1.19, p = 0.312
Fullness	1.60 (2.01)^a^	1.17 (1.79)^a^	3.10 (2.25)^b^	**F** = **4.83, p** = **0.012**
Appreciation	6.10 (1.83)	5.56 (1.76)	5.70 (2.11)	F = 0.46, p = 0.635
Food desire	2.90 (3.01)	4.89 (2.87)	4.50 (2.85)	F = 2.66, p = 0.079
BIS	14.06 (2.94)^a^	14.67 (2.43)^a^	12.15 (3.60)^b^	**F = 4.26, p = 0.019**
BAS	25.50 (5.18)	26.44 (5.01)	24.80 (4.51)	F = 0.55, p = 0.581
DEBQ				
Restrained	21.50 (6.64)^a^	26.89 (5.70)^b^	26.87 (5.06)^b^	**F** = **4.95, p** = **0.011**
External	29.70 (3.48)^a^	27.06 (3.44)^b^	32.85 (4.08)^c^	**F** = **11.29, p** = **0.00009**
Emotional	8.55 (3.07)^a^	10.11 (2.84)^a^	13.85 (3.12)^b^	**F** = **16.50, p** = **0.000003**
Binge Eating	10.90 (1.55)^a^	11.56 (3.17)^a^	14.40 (2.46)^b^	**F** = **10.13, p** =** 0.0002**
Food Awareness	19.11 (4.48)^a^	16.71 (2.83)^b^	14.79 (3.15)^c^	**F** = **6.80, p** = **0.002**

Different superscript indicate a significant difference after Bonferonni correction (p = 0.05).

**Table 3:** YFAS subscales for the lean and obese groups.

	Lean	Obesity		
		Low YFAS	High YFAS	
Substance taken in larger amount and for longer period than intended	20.00^a^	5.56^a^	75.00^b^	**χ^2^ = 23.06, p = 0.00001**
Persistent desire or repeated unsuccessful attempts to quit	100.00	94.44	100.00	χ^2^ = 0.10, n.s.
Much time/activity to obtain, use, recover	20.00^a^	22.22^a^	100.00^b^	**χ^2^ = 30.76, p < 0.00001**
Important social occupational, or recreational activities given up or reduced	5.00^a^	5.56^a^	65.00^b^	**χ^2^ = 24.39, p = 0.000005**
Use continues despite knowledge of adverse consequences	100.00	94.44	100.00	χ^2^ = 0.10, n.s.
Tolerance	100.00	94.44	100.00	χ^2^ = 0.10, n.s.
Characteristic withdrawal symptoms; substance taken to relieve withdrawal	10.00^a^	11.11^a^	85.00^b^	**χ^2^ = 31.47, p < 0.00001**
Use causes clinically significant impairment or distress	5.00^a^	0.00^a^	55.00^b^	**χ^2^ = 22.04, p = 0.00002**
Total YFAS	3.60 (1.05)^a^	3.50 (0.51)^a^	6.80 (0.77)^b^	**F = 104.18, p = 0.000001**

Radar image representing percentage of people exhibiting each food related symptom. The food addicted obese group (high YFAS) behaves differently from the lean and the non-food addicted obese group (low YFAS). The lean and non-food addicted group show exactly the same food related behaviour.

Different superscript indicate a significant difference (p = 0.05).

**Table 4:** Pearson Correlation between the different questionnaires.

	YFAS	Hunger	Satisfied	Fullness	Appreciation	Food desire	BIS	BAS	DEBQ Restrained	EBQ External	DEBQ Emotional	Binge Eating
*Whole goup*												
Food Awareness	−0.34*	0.18	0.02	−0.14	−0.10	−0.02	0.18	−0.26	0.33*	−0.18	−0.26	−0.22
Binge Eating	0.50**	−0.03	0.00	0.17	0.04	−0.08	−0.39**	−0.29*	−0.22	0.35*	0.43**	
DEBQ: Emotional	0.56***	−0.06	−0.04	0.11	0.20	−0.10	−0.55***	−0.19	0.10	0.54		
DEBQ External	0.55***	−0.09	0.07	0.13	0.00	0.04	−0.36*	−0.25	−0.09			
DEBQ Restrained	−0.03	0.05	−0.18	−0.20	−0.01	0.14	−0.21	−0.21				
BAS	−0.16	0.03	−0.10	−0.10	0.36*	−0.15	0.03					
BIS	−0.37*	−0.18	0.28	−0.04	−0.34*	0.27						
Food desire	0.08	−0.40**	0.50***	0.32*	−0.30*							
Appreciation	0.08	0.56***	−0.03	−0.07								
Fullness	0.42**	−0.21	0.55									
Satisfied	0.10	−0.18										
Hunger	−0.06											
*Low YFAS*												
Food Awareness	0.21	0.06	0.15	−0.16	−0.15	0.45	−0.24	−0.22	0.48	−0.01	−0.04	−0.10
Binge Eating	0.18	−0.03	−0.28	−0.18	−0.02	−0.06	−0.31	−0.49*	0.06	−0.15	−0.03	
DEBQ: Emotional	0.34	0.23	−0.17	−0.04	0.31	−0.05	−0.38	−0.30	0.42	0.38		
DEBQ External	0.13	0.10	0.15	−0.31	0.12	0.03	0.10	−0.14	−0.07			
DEBQ Restrained	0.38	−0.07	−0.29	−0.33	0.08	0.30	−0.54*	−0.21				
BAS	−0.22	−0.01	0.16	0.13	0.34	0.01	0.34					
BIS	−0.37	−0.37	0.54*	0.47*	−0.21	0.17						
Food desire	−0.13	−0.29	0.51*	0.04	−0.27							
Appreciation	0.28	0.63**	0.00	−0.02								
Fullness	−0.30	−0.33	0.56*									
Satisfied	−0.21	−0.20										
Hunger	0.43											
*High YFAS*												
Food Awareness	−0.51*	−0.05	0.33	0.18	−0.35	−0.17	0.26	−0.24	0.08	−0.26	−0.20	−0.20
Binge Eating	0.56*	−0.16	0.20	0.11	0.07	0.06	−0.28	−0.05	−0.59*	0.26	0.59**	
DEBQ: Emotional	0.37	0.04	−0.07	−0.17	0.28	−0.25	0.47	−0.01	−0.18	0.31		
DEBQ External	0.34	−0.40	0.00	−0.08	−0.06	0.19	−0.24	−0.09	−0.34			
DEBQ Restrained	−0.47	0.40	−0.03	−0.05	0.04	−0.06	−0.14	−0.11				
BAS	0.04	0.15	−0.31	0.22	0.50*	−0.34	−0.15					
BIS	−0.06	−0.27	0.16	−0.02	−0.50*	0.34						
Food desire	0.39	−0.47*	0.44	0.61**	−0.39							
Appreciation	0.12	0.54*	−0.02	−0.17								
Fullness	0.24	−0.38	0.56*									
Satisfied	0.29	−0.16										
Hunger	−0.37											

*p < 0.05, **p < 0.01, ***p < 0.001.
